# Preprotachykinin A is expressed by a distinct population of excitatory neurons in the mouse superficial spinal dorsal horn including cells that respond to noxious and pruritic stimuli

**DOI:** 10.1097/j.pain.0000000000000778

**Published:** 2016-11-30

**Authors:** Maria Gutierrez-Mecinas, Andrew M. Bell, Alina Marin, Rebecca Taylor, Kieran A. Boyle, Takahiro Furuta, Masahiko Watanabe, Erika Polgár, Andrew J. Todd

**Affiliations:** a Institute of Neuroscience and Psychology, University of Glasgow, Glasgow, United Kingdom; b School of Veterinary Medicine, University of Glasgow, Glasgow, United Kingdom; c Department of Morphological Brain Science, Graduate School of Medicine, Kyoto University, Kyoto, Japan; d Department of Anatomy, Hokkaido University School of Medicine, Sapporo, Japan

**Keywords:** Substance P, Tac1, Excitatory interneuron, Pain, Itch

## Abstract

The superficial dorsal horn, which is the main target for nociceptive and pruritoceptive primary afferents, contains a high density of excitatory interneurons. Our understanding of their roles in somatosensory processing has been restricted by the difficulty of distinguishing functional populations among these cells. We recently defined 3 nonoverlapping populations among the excitatory neurons, based on the expression of neurotensin, neurokinin B, and gastrin-releasing peptide. Here we identify and characterise another population: neurons that express the tachykinin peptide substance P. We show with immunocytochemistry that its precursor protein (preprotachykinin A, PPTA) can be detected in ∼14% of lamina I-II neurons, and these are concentrated in the outer part of lamina II. Over 80% of the PPTA-positive cells lack the transcription factor Pax2 (which determines an inhibitory phenotype), and these account for ∼15% of the excitatory neurons in this region. They are different from the neurotensin, neurokinin B, or gastrin-releasing peptide neurons, although many of them contain somatostatin, which is widely expressed among superficial dorsal horn excitatory interneurons. We show that many of these cells respond to noxious thermal and mechanical stimuli and to intradermal injection of pruritogens. Finally, we demonstrate that these cells can also be identified in a knock-in Cre mouse line (Tac1^Cre^), although our findings suggest that there is an additional population of neurons that transiently express PPTA. This population of substance P–expressing excitatory neurons is likely to play an important role in the transmission of signals that are perceived as pain and itch.

## Abstract 2

Expression of the substance P precursor preprotachykinin A defines a distinct population of superficial dorsal horn excitatory neurons, many of which respond to noxious or pruritic stimuli.

## 1. Introduction

The superficial dorsal horn (laminae I-II) is innervated by primary afferents that detect noxious and pruritic stimuli. This information is modulated by local neuronal circuits before being transmitted by projection neurons to the brain, where it contributes to conscious perception. We still have only a limited understanding of sensory processing in the dorsal horn, and this is largely due to the very complex organisation of its constituent neurons and the synaptic circuits to which they contribute.^5,22,78^

The great majority of neurons in laminae I-II are interneurons, with axonal projections that remain within the spinal cord, and these can be divided into 2 broad functional classes: excitatory (glutamatergic) neurons and inhibitory neurons that use γ-aminobutyric acid (GABA) and/or glycine as their principal transmitter.^5,70,78,91,93^ Anatomical and electrophysiological studies have revealed that there is considerable heterogeneity within both these classes, and there have therefore been numerous attempts to define functional populations among both the excitatory and inhibitory populations. However, although certain morphological classes can be recognised among these cells,^23,89^ many of them cannot be classified based solely on the basis of somatodendritic morphology.^20,36,91^ An alternative approach is to use neurochemical markers. We have identified 4 largely nonoverlapping neurochemical populations that account for over half of the inhibitory interneurons in this region and shown that these differ in their responses to noxious stimuli and synaptic connections, as well as in their dependence on transcription factors during development.^41,64,78^

There is less information available concerning the organisation of excitatory interneurons in this region, although we recently reported that 3 nonoverlapping populations could be identified by their expression of neurotensin, neurokinin B (NKB), and gastrin-releasing peptide (GRP).^26^ These cells accounted for nearly 40% of the excitatory neurons in laminae I-II, and all 3 of these neuropeptides overlapped extensively with somatostatin, which is expressed by the majority of glutamatergic neurons in this region.^16,26^ During the course of that study, we found evidence that an additional population of excitatory neurons might be defined by the expression of substance P (SP), which is cleaved from a precursor protein, preprotachykinin A (PPTA), coded by the gene *Tac1*. Although in situ hybridisation studies have identified cells with PPTA mRNA in the superficial dorsal horn,^86,88^ the level of SP in their cell bodies is normally below the threshold for detection with immunocytochemistry. Substance P–expressing neurons elsewhere in the central nervous system have been revealed with antibodies against PPTA,^19,40^ and we therefore tested whether this approach could be used to reveal these cells in the dorsal horn. We went on to characterise the cells and test their responsiveness to different noxious and pruritic stimuli. Finally, we used a genetically modified mouse in which Cre recombinase is inserted into the *Tac1* locus (Tac1^Cre^) to show that SP-expressing neurons can also be revealed by intraspinal injection of adenoassociated virus (AAV) coding for a Cre-dependent form of enhanced green fluorescent protein (eGFP).

## 2. Methods

Experiments were approved by the Ethical Review Process Applications Panel of the University of Glasgow and were performed in accordance with the U.K. Animals (Scientific Procedures) Act 1986.

### 2.1. Characterisation of preprotachykinin A–immunoreactive neurons

Five wild-type C57Bl/6 mice (either sex, 18-27 g) and 3 transgenic mice (either sex, 20-31 g) in which eGFP was expressed under the control of GRP promoter (GRP-eGFP)^3,27,51,73^ were deeply anaesthetised with pentobarbitone (30 mg intraperitoneally) and perfused through the left ventricle with 4% formaldehyde in phosphate buffer (PB). The GRP-eGFP mice were used to allow identification of GRP-expressing neurons, since GRP itself cannot be detected in neuronal cell bodies, and it has been shown that over 90% of eGFP-positive cells in this line have detectable GRP mRNA.^73^

Spinal cord segments L3 to L5 were postfixed for 2 hours and cut into 60-μm-thick transverse sections with a vibrating blade microtome (Leica VT1200 or VT1000S). The sections were processed for immunocytochemistry as described previously.^20^ They were incubated in primary antibodies for 3 days and in secondary antibodies for 1 day, in both cases at 4°C. All antibodies were diluted in phosphate-buffered saline that contained 0.3% Triton-X100. Details of the sources and concentrations of primary antibodies are shown in Table 1. Species-specific secondary antibodies (Jackson Immunoresearch, West Grove, PA) were raised in donkey and conjugated to Alexa 488, Rhodamine Red, Alexa 647, Pacific Blue, or biotin. In all cases, the PPTA antibody was detected with a biotinylated secondary antibody and revealed with a tyramide signal amplification (TSA) method (TSA kit tetramethyl-rhodamine NL702; PerkinElmer Life Sciences, Boston, MA) as described previously.^26^ In some cases, sections were incubated in DAPI to reveal cell nuclei. Sections were then mounted in antifade medium and stored at −20°C. They were scanned with a Zeiss LSM710 confocal microscope equipped with Argon multiline, 405 nm diode, 561 nm solid state, and 633 nm HeNe lasers. Scans were obtained through ×40 or ×63 oil immersion lenses (numerical apertures 1.3 and 1.4, respectively), with the confocal aperture set to 1 Airy unit or less.

**Table 1:**
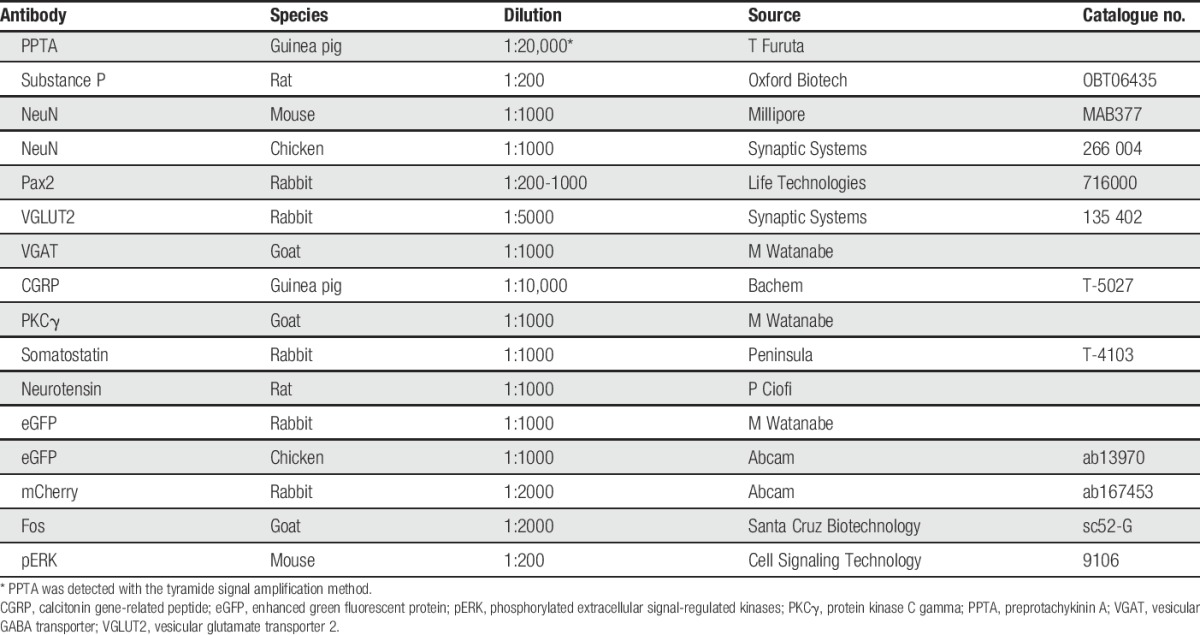
Antibodies used in this study.

Sections from wild-type mice that had been reacted to reveal PPTA, SP, and NeuN were initially examined to reveal the relationship between SP and PPTA and to establish whether PPTA could be detected in neuronal cell bodies.

Sections from 3 wild-type mice that had been reacted with PPTA, Pax2, and NeuN antibodies and incubated in DAPI were then used to determine the proportion of neurons in laminae I-III that contained PPTA and whether any of these expressed Pax2, which is a marker for inhibitory interneurons.^11,18,41^ Two sections from each mouse were selected for analysis, and one side was scanned to generate z-stacks of 25 to 35 optical sections at 1 μm z-spacing, covering the entire cross-sectional area of laminae I-III. These were analysed with Neurolucida for Confocal software (MBF Bioscience, Williston, VT). We used a modification of the optical disector method^74^ described previously^26,63^ to obtain an unbiased sample of neurons in laminae I-II and lamina III. The borders between laminae II/III and III/IV were identified by the relatively low packing densities of neurons in the inner half of lamina II (IIi)^82^ and in lamina IV.^26^ The distance between reference and lookup sections for the disector sample was between 10 and 15 μm. Neuronal nuclei (defined by the presence of NeuN and DAPI staining) with their bottom surface between the reference and lookup sections were selected and plotted onto an outline of the dorsal horn. In all cases, we examined all optical sections between the reference and lookup and added the locations of any cells that were entirely contained between these 2 optical sections.^26^ The channels corresponding to PPTA and Pax2 were then viewed, and the presence or absence of each type of immunoreactivity was noted for the selected neurons. In this way, we determined the proportion of all neurons in laminae I-II and in lamina III that were PPTA and/or Pax2 immunoreactive.

Since we found that some PPTA-expressing cells were Pax2 positive and were therefore inhibitory interneurons, we looked for evidence that SP could be detected in axon terminals that expressed the vesicular GABA transporter (VGAT). This was achieved by scanning sections from 3 wild-type mice that had been reacted with a combination of antibodies against SP, calcitonin gene-related peptide (CGRP), VGAT, and the vesicular glutamate transporter 2 (VGLUT2). The VGLUT2 and CGRP antibodies were included to allow identification of axons belonging to excitatory interneurons (which show strong VGLUT2 immunoreactivity), as well as SP-containing primary afferents (which also express CGRP).^39,45,55,83^

We next investigated the relationship of PPTA to other markers that define neurochemical populations of excitatory interneurons^26^: somatostatin, protein kinase C (PKC) γ, neurotensin, and GRP. For this analysis, sections from wild-type or GRP-eGFP mice were reacted with the following antibody combinations: (1) PPTA, somatostatin, and NeuN; (2) PPTA, PKCγ, Pax2, and NeuN; (3) PPTA, neurotensin, PKCγ, and NeuN; and (4) PPTA, GFP, and NeuN (this reaction was performed on sections from GRP-eGFP mice). In each case, tissue from 3 mice was reacted and analysed. For the first combination (PPTA, somatostatin, and NeuN), we selected 2 sections from each mouse and these were scanned with the confocal microscope and analysed using the disector method, as described above. In the cases of PKCγ, neurotensin, and GRP-eGFP, we found that none, or very few, of the PPTA cells were included in these populations, and we therefore counted sections in confocal z-stacks without using a stereological method.

We could not test directly for colocalisation of PPTA and preprotachykinin B (PPTB, the precursor for NKB), since the antibodies that we have against both these tachykinin precursors were raised in guinea pig.^62^ We therefore used an indirect approach by looking for colocalisation in axon terminals derived from these cells. We had previously noted that very few VGLUT2-containing (glutamatergic) boutons in the superficial dorsal horn were immunoreactive for both SP and PPTB,^27^ suggesting that PPTA and PPTB are expressed by largely nonoverlapping populations. To provide quantitative information, we reexamined sections used in that study.^27^ These were from GRP-eGFP mice and had been reacted to reveal VGLUT2, SP, PPTB, and eGFP (as shown in Fig. 10 of Ref. 27). Two sections each from 2 mice were scanned to generate z-stacks through laminae I-II and the dorsal part of lamina III. These were analysed with Neurolucida for Confocal. Initially, the channel corresponding to PPTB was switched off, and 50 SP-immunoreactive boutons with high levels of VGLUT2 were selected, as these are likely to correspond to axons of SP-expressing glutamatergic dorsal horn neurons.^79^ Boutons were selected throughout the dorsoventral extent of the region scanned. The PPTB channel was then switched on, and the presence or absence of PPTB immunostaining was noted for each selected bouton. Each section was then reanalysed by selecting 50 boutons that were PPTB and VGLUT2 immunoreactive and then revealing the SP channel and noting the presence or absence of staining in each bouton.

### 2.2. Responses of preprotachykinin A–expressing cells to noxious and pruritic stimulation

Nineteen C57Bl/6 mice of either sex (18-29 g) received either a noxious or a pruritic stimulus prior to perfusion fixation, and tissue from these animals was used to test for expression of the transcription factor Fos,^35^ or phosphorylation of extracellular signal-regulated kinases (ERK),^38^ in PPTA-immunoreactive cells. Most of the stimuli were applied while mice were under brief isoflurane anaesthesia, and they were then allowed to recover for the remainder of the 2-hour survival period. The following stimuli were applied in this way: (1) noxious heat (immersion of the hind paw in water at 52°C for 15 seconds [n = 3 mice]), (2) intraplantar injection of 10 μL of 0.125% capsaicin^64^ (n = 4), (3) intradermal injection of histamine (100 μg in 10 μL PB) into the calf (n = 4), or (4) intradermal injection of chloroquine (50 μg in 10 μL PB) into the calf (n = 4). In all cases, stimuli were applied to the left hind limb. For the animals that received histamine or chloroquine, the skin over the calf was shaved on the day before stimulation and Elizabethan collars were applied at the time of shaving and left on for the duration of the experiment to prevent the animals from scratching or biting the injected areas.^3^ The mice were re-anaesthetised with pentobarbitone and perfused with fixative (as described above) 2 hours after the stimulus. Fewer mice (n = 3) were tested with the noxious heat because this stimulus resulted in considerably more Fos cells per section than the other stimuli.

In the case of noxious mechanical stimulation, we have found that immunostaining for phosphorylated ERK (pERK) is more reliable than Fos immunocytochemistry for detecting activated cells.^36,64^ pERK appears very rapidly after noxious stimulation,^36^ and these experiments were therefore performed under terminal general anaesthesia. Four mice were anaesthetised with 10% urethane intraperitoneally, and skin on the lateral aspect of the left calf was pinched with forceps at 5 separate locations for 5 seconds each. The mice were perfused with fixative 5 minutes after the last stimulus.

Transverse sections through the L3 or L4 segments of these mice were reacted to reveal PPTA, Pax2, and NeuN, together with either Fos or pERK. Between 4 and 8 sections that contained numerous Fos- or pERK-positive neurons were identified from each experiment and scanned through the ×40 lens with 1 or 2 μm z-separation to include the mediolateral extent of the area in which Fos- or pERK-positive cells were present.^3^ The resulting z-stacks were analysed with Neurolucida using the modified disector method described above. We initially drew parallel lines at right angles to the laminar boundaries to define the region of superficial dorsal horn that contained a high density of Fos or pERK cells as described previously.^3^ We then examined the channel corresponding to NeuN and identified all neurons in laminae I-II that were between these 2 lines and had their lower surface between the reference and lookup sections. The remaining channels were then viewed, and for each of the selected neurons, we determined whether they were immunoreactive for PPTA, Pax2, and either Fos or pERK.

We have previously reported that intradermal injection of vehicle into the calf does not result in significant Fos labelling in the dorsal horn,^3^ and we have found that this is also the case for intraplantar injection of vehicle (EP, AJT unpublished observations).

### 2.3. Injection of Cre-dependent reporter virus into the Tac1^Cre^ mouse

In order to verify the expression of PPTA with an independent approach, we used mice in which Cre recombinase was knocked into the *Tac1* locus^29^ (Tac1-IRES2-Cre-D; The Jackson Laboratory, Bar Harbor, ME; Stock number 021877). These mice, referred to as Tac1^Cre^, were bred with a reporter line Ai9 (Jackson Laboratory; Stock number 007909), in which Cre-mediated excision of a STOP cassette drives expression of the red fluorescent protein tdTomato (tdTom). The resulting offspring (Tac1^Cre^;Ai9) should have tdTom in all neurons that have expressed *Tac1* at any stage during development. Two adult female Tac1^Cre^;Ai9 mice (18, 20 g) were anaesthetised with isoflurane and received intraspinal injections of an AAV (serotype 1) coding for a Cre-dependent form of eGFP (AAV.flex.eGFP; Penn Vector Core, Philadelphia, PA). This virus encodes an inverted sequence for eGFP between pairs of heterotypic LoxP sites with antiparallel orientation.^2^ In infected cells that express Cre at the time of injection, there will be permanent reversal of the coding sequence, resulting in expression of eGFP. Injections were made at 2 sites in the dorsal horn, based on the method described by Foster et al.^18^ The vertebrae T12 and L1 were identified and clamped with spinal adaptors attached to a stereotaxic frame. A small slit was made in the dura on either side of the T13 vertebra, and injections were made through a glass micropipette (tip diameter 40 μm) into the dorsal horn on the right side at a depth of 300 μm below the dorsal surface. A volume of 300 nL (containing 1.72 × 10^9^ gene copies) was made at each injection site at a rate of 30 nL/min. The wound was closed, and animals were allowed to recover with appropriate analgesia. After an 8-day survival period, they were re-anaesthetised and perfused with fixative as described above. The rationale for this approach is that among neurons infected by AAV.flex.eGFP in the region of the injection site, those that continue to express PPTA should generate eGFP and will therefore contain both fluorescent proteins. In contrast, neurons that expressed PPTA only transiently during earlier development will synthesise tdTom but not eGFP.

Transverse sections from the L3 segment were cut into 3 bottles, which were processed for immunocytochemistry as follows: (1) anti-mCherry and chicken anti-NeuN, (2) Pax2 and PKCγ, and (3) PPTA. The anti-mCherry, which also detects tdTom, was used in the first reaction to amplify the tdTom signal, and these sections were subsequently stained with DAPI. The chicken (rather than the mouse) NeuN antibody was used in this case because we have found that immunoreactions involving mouse antibodies show high background staining in animals that have received intraspinal injections, presumably due to the local immune response. In the third reaction, the PPTA was revealed with a TSA method involving the far-red dye Cy5 (TSA kit NL705; PerkinElmer Life Sciences).

In order to determine the proportions of neurons that contained tdTom and eGFP, we selected 3 sections per mouse from the first reaction (mCherry, NeuN, and DAPI) that had numerous eGFP^+^ cells. These were scanned through the ×40 lens to include the area containing the eGFP cells. Z-stacks (1 μm separation) were acquired and analysed with the disector method as described above. The distance between reference and lookup sections was 10 μm. Neurons in laminae I-II for which the bottom surface of the nucleus was included in the disector were first identified. The channels corresponding to eGFP and tdTom were then viewed, and the presence or absence of each fluorescent protein in the selected neurons was recorded.

During the analysis of PPTA-immunoreactive neurons, we found that these included some Pax2-positive (inhibitory) neurons but that they were very seldom immunoreactive for PKCγ, which is expressed by a distinct subset of excitatory interneurons.^26,61^ We therefore analysed 2 sections from the second antibody combination (Pax2 and PKCγ) from each mouse. These were scanned with the ×40 lens (1 μm z-separation) through the full thickness of the section to include the region that contained eGFP cells. The resulting z-stacks were analysed with Neurolucida, and all neurons that contained either eGFP or tdTom were identified. The channels corresponding to Pax2 and PKCγ were then viewed, and the presence or absence of each type of staining was recorded for the selected cells. Although a stereological technique was not used for this analysis, the error due to oversampling of cells at the section surfaces^24^ is likely to be small, since the section thickness (60 μm) was much greater than the typical diameter of these neurons.

To test for the presence of PPTA in cells that were either tdTom or tdTom/eGFP labelled, 2 or 3 sections per mouse that contained numerous eGFP cells were selected from the third immunoreaction. These were scanned through the ×40 lens with a 1 μm z-separation. Initially, the channels corresponding to tdTom and PPTA immunoreactivity were viewed. From each animal, 50 tdTom^+^ cells that were PPTA immunoreactive were identified. The eGFP channel was then viewed, and the presence or absence of eGFP was recorded for each selected cell.

### 2.4. Antibody characterisation

The PPTA antibody was raised against a sequence of 15 amino acids (residues 98-112 of α-PPTA) and recognises PPTA, but not SP or neurokinin A.^44^ The monoclonal SP antibody detects the C-terminal 5 to 8 amino acids of SP and does not appear to recognise NKB.^13,50,62^ The monoclonal NeuN antibody was raised against cell nuclei extracted from mouse brain and reacts with a protein specific for neurons,^53^ which was subsequently identified as the splicing factor Fox-3.^42^ The chicken NeuN antibody was raised against a recombinant protein corresponding to amino acids 1 to 97 of mouse Fox-3. The antibody against Pax2 was raised against amino acids 188 to 385 of the mouse protein. The VGLUT2 and VGAT antibodies were raised against amino acids 510 to 582 of rat VGLUT2 and amino acids 31 to 112 of mouse VGAT, respectively, and both detect single protein bands of the appropriate molecular weight.^52,77^ The CGRP antibody detects both α and β forms of the peptide (manufacturer's specification). The PKCγ antibody was raised against amino acids 684 to 697 of the mouse protein and detects a single band at 75 kDa.^57^ The somatostatin antibody is reported to show 100% cross-reactivity with somatostatin-28 and somatostatin-25, but none with SP or neuropeptide Y, and staining is blocked by preincubation with somatostatin.^66^ Staining with the rat anti-neurotensin antibody is identical to that seen with a well-characterised rabbit antibody and is blocked by preincubation with the peptide.^65^ The eGFP antibodies were raised against recombinant full-length eGFP, and their staining matches that of native eGFP fluorescence. The mCherry antibody was raised against full-length recombinant protein and also recognises tdTom. The Fos antibody was raised against a peptide corresponding to the N-terminus of human Fos, whereas the pERK antibody is specific for ERK1 and ERK2 that are dually phosphorylated at Thr202 and Tyr204 sites. Specificity of the Fos and pERK antibodies was shown by the restriction of staining to neurons in somatotopically appropriate areas, after noxious or pruritic stimulation.

### 2.5. Statistics

Data were formatted into 2 individual 2  ×  2 contingency tables for each of the animals that received noxious or pruritic stimulation, with rows corresponding to the presence or absence of eGFP or Pax2 and columns to the presence or absence of Fos or pERK. To determine whether there was a consistent difference in the proportions across the animals for the different cell populations, we used the Mantel–Haenszel analysis.^3^ In all cases, Breslow–Day testing was performed to test the assumption that the odds ratio was the same across animals.^3^

## 3. Results

### 3.1. Preprotachykinin A–expressing neurons and their neurotransmitter phenotype

**Figure 1.**
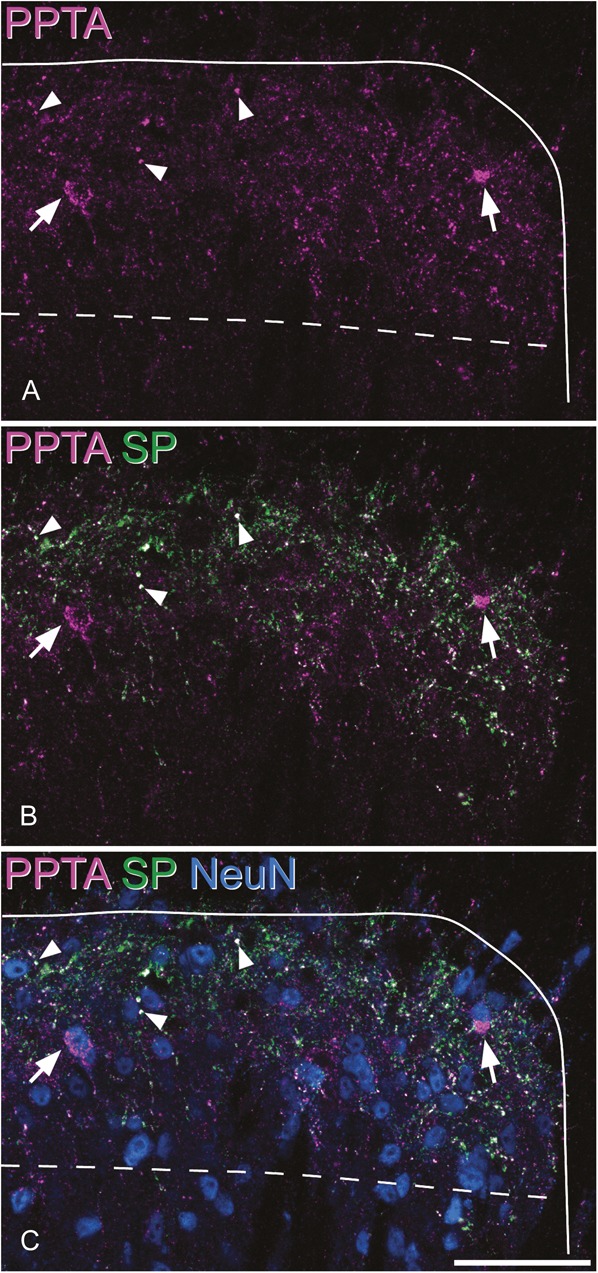
Immunostaining for preprotachykinin A (PPTA), substance P (SP), and NeuN in the mouse dorsal horn. (A) PPTA immunoreactivity (magenta) appears as fine punctate structures, many of which are axons (3 indicated with arrowheads), as well as large clumps (arrows) that represent staining in the perikaryal cytoplasm of certain neurons. (B) SP (green) is colocalised with PPTA in the axons, but not in the perikaryal cytoplasm. (C) Staining for the neuron-specific protein NeuN confirms that the clumps of PPTA shown with the arrows are in the cell bodies of 2 of the neurons. The solid line indicates the outline of the gray matter, and the dashed line shows the border between laminae II and III. The images are projected from 2 optical sections at 1 μm z-spacing. Scale bar = 50 μm.

Immunostaining for PPTA was concentrated in the superficial laminae (I-II) and present at lower levels in the deeper parts of the dorsal horn. In sections reacted with antibodies against PPTA and SP, PPTA staining was seen in small profiles scattered through the neuropil, and these were frequently also SP immunoreactive (Fig. 1A and B). The PPTA was also detected in the cell bodies of certain neurons, which were identified by the presence of NeuN (Fig. 1C). This cell body labelling with the PPTA antibody was restricted to the perikaryal cytoplasm and was never colocalised with SP immunoreactivity. The small profiles that were both PPTA and SP immunoreactive are likely to be axon terminals, whereas the cytoplasmic staining presumably corresponds to PPTA in the endoplasmic reticulum and/or Golgi apparatus of PPTA-expressing neurons. Neurons containing PPTA were present throughout laminae I-III and were occasionally seen in deeper laminae. The axonal staining for PPTA was generally restricted to the superficial parts of the sections, presumably due to the limited penetration of antibodies in fine axonal processes. In contrast, the cell body labelling was present throughout the depth of the sections, and there was no obvious difference in the frequency of immunoreactive neurons at different depths in the section.

**Figure 2.**
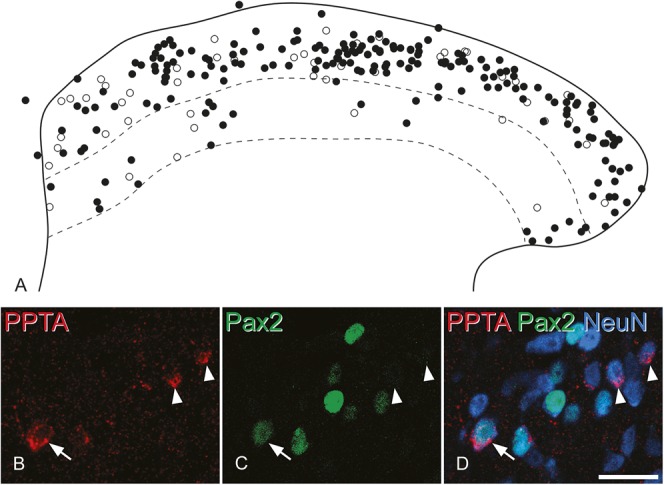
The distribution of excitatory and inhibitory preprotachykinin A (PPTA) cells. (A) Plot of the distribution of all PPTA-immunoreactive cells obtained from the stereological analysis of 3 mice (2 sections each) showing the location of Pax2-negative (excitatory) cells as filled circles and Pax2-positive (inhibitory) cells as open circles. The solid line represents the outline of the gray matter, and the upper and lower dashed lines represent the borders between laminae II-III and III-IV, respectively. (B–D) Examples of Pax2-negative and positive cells seen in confocal images. These have been scanned to reveal PPTA (red), Pax2 (green), and NeuN (blue). The arrow indicates a PPTA-immunoreactive cell that was Pax2 positive, whereas the arrowheads show 2 PPTA-immunoreactive cells that lack Pax2. The images are projections of 3 optical sections at 2 μm z-spacing. Scale bar = 20 μm.

Quantitative analysis revealed that PPTA-immunoreactive cells accounted for 14% of the neurons in laminae I-II and 4% of those in lamina III (Table 2). In sections that had been stained for PPTA and Pax2, we found that although the majority of PPTA^+^ neurons were Pax2 negative, there were also PPTA^+^/Pax2^+^ cells scattered throughout laminae I-III (Fig. 2), indicating that some of the PPTA-expressing cells were inhibitory interneurons. Pax2 immunoreactivity was observed in approximately 19% of the PPTA^+^ neurons in laminae I-II and in 21% of those in lamina III (Table 2). The proportions of all neurons that were Pax2 in these sections were 25% for laminae I-II and 40% for lamina III, and these values are very close to our previous estimates of the proportion of neurons in these regions that are GABA and/or glycine immunoreactive in the mouse spinal cord (25.8% for laminae I-II and 37.6% for lamina III).^60^ This provides further support for the suggestion that immunostaining for Pax2 reveals virtually all inhibitory interneurons in this region.^18,41^ Since the remaining neurons (74.2% of those in laminae I-II and 62.4% of those in lamina III) are presumably glutamatergic,^60^ the present results suggest that PPTA-immunoreactive neurons account for ∼15% of glutamatergic (excitatory) neurons in laminae I-II and 5% of those in lamina III (Table 2).

**Table 2:**
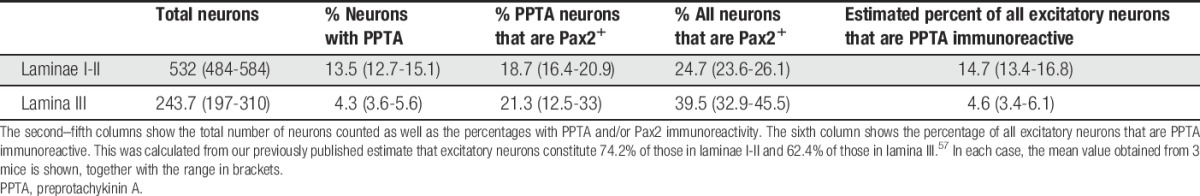
The proportions of neurons that express PPTA or Pax2.

**Figure 3.**
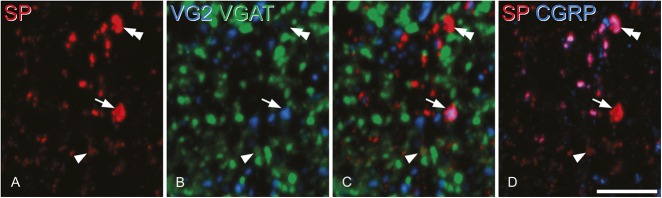
Immunostaining for substance P(SP), calcitonin gene-related peptide (CGRP), and vesicular neurotransmitter transporters in lamina II of the dorsal horn. (A) A field that contains several SP-immunoreactive profiles (red). (B) The same field scanned to reveal vesicular glutamate transporter 2 (VG2, blue) and vesicular GABA transporter (VGAT, green). (C) A merged image showing SP, VG2, and VGAT. (D) The same field scanned to reveal SP (red) and CGRP (blue). Comparison of these images shows that some SP-immunoreactive profiles are strongly labelled for VG2 and are likely to correspond to axons of excitatory preprotachykinin A–expressing neurons (one indicated with arrow), whereas others are immunoreactive for CGRP and represent central terminals of peptidergic primary afferents (one shown with double arrowhead). Occasionally, VGAT-positive profiles with very weak SP immunoreactivity are present, and one of these is indicated with an arrowhead. All images are projections of 3 optical sections at 0.3 μm z-spacing. Scale bar = 5 μm.

Substance P–containing axons in the dorsal horn are thought to originate predominantly from 2 sources: primary afferents (in which SP is colocalised with CGRP^39,45,83^) and local neurons. We have previously reported that in the rat, all SP-containing axonal boutons that lacked CGRP were VGLUT2 immunoreactive,^79^ which would suggest that SP (and therefore PPTA) was only expressed by glutamatergic dorsal horn neurons. We were therefore surprised to find that significant numbers of PPTA-immunoreactive neurons in laminae I-III were Pax2 positive (inhibitory). To test whether this discrepancy resulted from a species difference, we examined sections of mouse spinal cord that had been reacted with antibodies against SP, CGRP, VGLUT2, and VGAT. We found that, as in the rat, boutons with strong SP immunoreactivity were invariably either CGRP immunoreactive (with weak or undetectable levels of VGLUT2^79^) or showed strong VGLUT2 immunostaining and were CGRP negative. However, in each of laminae I-III, we occasionally observed extremely weak staining for SP in VGAT-immunoreactive boutons (Fig. 3). This suggests that although PPTA is present in some inhibitory interneurons in these laminae, the level of SP in their axon terminals is very low.

### 3.2. Relation of preprotachykinin A–expressing excitatory neurons to other subpopulations

**Figure 4.**
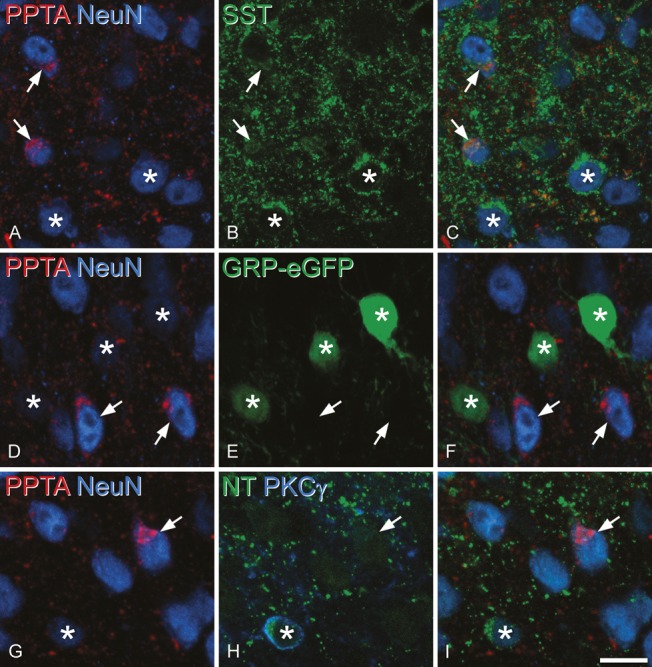
Neurochemical characterisation of preprotachykinin A (PPTA)–immunoreactive cells. (A–C) Immunostaining for PPTA (red), NeuN (blue), and somatostatin (SST, green) reveals neurons with both PPTA and somatostatin in their perikaryal cytoplasm (2 indicated with arrows), as well as cells that are only positive for somatostatin (2 marked with asterisks). (D–F) A section from a gastrin-releasing peptide enhanced green fluorescent protein (eGFP) mouse stained for eGFP (green), PPTA (red), and NeuN (blue). The PPTA-immunoreactive cells (2 shown with arrows) were completely separate from eGFP^+^ cells (3 marked with asterisks). (G–I) A section immunostained to reveal PPTA (red), NeuN (blue), neurotensin (NT, green), and protein kinase C (PKC) γ (blue). (G) The section has been scanned to reveal PPTA and NeuN and shows a cell that is PPTA immunoreactive (arrow), as well as several that are PPTA negative (one of which is marked with an asterisk). (H) The same field scanned to reveal neurotensin and PKCγ. The cell marked with the arrow is negative for both markers. The cell with the asterisk is immunoreactive for both PKCγ (which labels the plasma membrane) and neurotensin (which is in the perikaryal cytoplasm). (I) A merge of PPTA, NeuN, and neurotensin confirms that PPTA and neurotensin are in different neurons. Images in (A–C) are projections of 4 optical sections at 0.5 μm z-spacing; those in (D–F) are projections of 2 optical sections 1 μm apart; those in (G–I) are projections of 3 optical sections at 0.5 μm z-spacing. Scale bar = 10 μm.

In the sections reacted with antibodies against PPTA, somatostatin, and NeuN, we analysed cells in laminae I-II, since somatostatin is virtually restricted to excitatory (glutamatergic) neurons in this region, whereas the peptide is expressed by both inhibitory and excitatory interneurons in lamina III.^16,66,87,88^ Between 370 and 596 neurons in laminae I-II were analysed in the 3 mice. Within this sample, 13.2% (range 12.4%-14%) were PPTA immunoreactive, whereas 44.8% (42.5%-46.6%) were immunoreactive for somatostatin. Many cells containing both types of immunoreactivity were observed (Fig. 4A–C), and these accounted for 57.4% (range 54.9%-62.2%) of the PPTA-positive neurons. Since 19% of PPTA-positive neurons in this region are Pax2^+^ (inhibitory) and somatostatin is restricted to excitatory neurons in this region, we estimate that 71% of the PPTA^+^ excitatory neurons are somatostatin immunoreactive.

In the sections from the 3 GRP-eGFP mice, we identified a mean of 151 (range 109-219) eGFP-positive cells and 155 (range 111-196) PPTA-immunoreactive cells in laminae I-III and found that none of these were double labelled (Fig. 4D–F).

In sections reacted with PPTA, PKCγ, and Pax2 antibodies, we identified between 109 and 132 (mean 117) PKCγ^+^ cells and between 39 and 44 PPTA^+^/Pax2-negative cells (mean 41.7) in laminae I-III (n = 3 mice). Although there was some overlap in the distribution of these 2 populations in laminae IIi-III, none of these cells were double labelled. We have previously shown that 90% of neurotensin-expressing cells are PKCγ^+26^ and would therefore not express PPTA. For this reason, we examined neurotensin-immunoreactive cells that lacked PKCγ in the sections that had been reacted with PPTA, neurotensin, PKCγ, and NeuN antibodies. We identified 61 of these cells (17-26 in each mouse) and found that only one of them was PPTA immunoreactive. An example of PPTA staining in a section reacted for PPTA, neurotensin, and PKCγ is shown in Figure 4G–I.

In the sections that had been reacted for PPTB, SP, and VGLUT2,^27^ we found minimal coexistence of PPTB and SP in VGLUT2-immunoreactive boutons. The majority (98.5%, range 98%-99%) of the selected SP-immunoreactive boutons were negative for PPTB, whereas the same proportion (range 97%-100%) of PPTB-immunoreactive boutons lacked staining for SP.

Taken together, these results indicate that PPTA is not expressed to a significant extent in excitatory interneurons that express GRP, NKB, neurotensin, or PKCγ. However, it overlaps extensively with somatostatin, such that the majority (∼70%) of PPTA-positive excitatory neurons contain somatostatin.

### 3.3. Expression of Fos and phosphorylated extracellular signal-regulated kinases after noxious or pruritic stimulation

**Figure 5.**
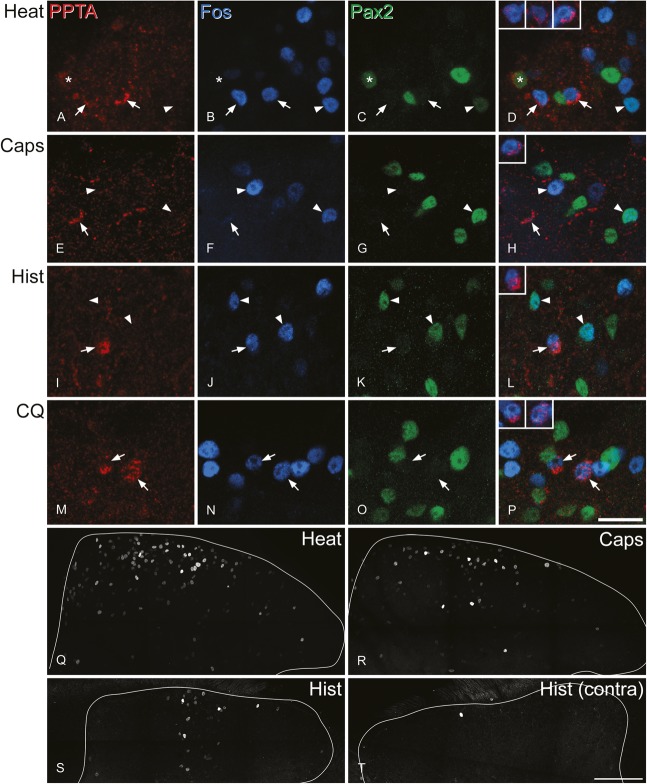
Fos immunoreactivity after noxious or pruritic stimulation. (A–D, E–H, I–L, and M–P) are from mice that were stimulated with noxious heat, capsaicin, histamine, or chloroquine, respectively. In each case, the same field from superficial dorsal horn is shown stained for preprotachykinin A (PPTA) (red), Fos (blue), and Pax2 (green), together with a merged image. The insets in (D, H, L, and P) show staining for PPTA (red) and NeuN (blue) in the marked PPTA-immunoreactive cells. (A–D) The noxious stimulus resulted in numerous Fos^+^ neurons. This field contains 2 PPTA-immunoreactive/Pax2-negative cells that have Fos-immunoreactive nuclei (arrows). In addition, a PPTA-negative cell that is positive for both Pax2 and Fos is shown with an arrowhead, and a PPTA-positive/Pax2-positive cell that lacks Fos is indicated with an asterisk. (E–H) Relatively few of the PPTA^+^ excitatory (Pax2-negative) cells showed Fos after capsaicin injection. This field shows one of these cells that lacks Fos (arrow). Two Fos-immunoreactive cells are indicated with arrowheads. The one on the left is negative for Pax2 while that on the right is Pax2^+^. (I–L) This field from a histamine-injected mouse shows a PPTA^+^ excitatory (Pax2-negative) cell that contains Fos (arrow). In addition, 2 PPTA-negative cells that are immunoreactive for both Fos and Pax2 are indicated (arrowheads). (M–P) After stimulation with chloroquine, several Fos-immunoreactive neurons are visible, and 2 of these (marked with arrows) are PPTA-immunoreactive excitatory (Pax2-negative) neurons. Images are projections of 3 (E–H), 5 (A–D), or 4 (I–P) optical sections at 1 μm z-spacing. (Q–S) Show lower magnification views of the ipsilateral dorsal horn from mice treated with noxious heat, capsaicin, or histamine (the same sections as those in A–L) scanned to reveal Fos. (T) Shows the contralateral side of the histamine-treated animal. Scale bars: A-P = 20 μm; Q-T = 100 μm.

After noxious heat or the injection of capsaicin or pruritogens (chloroquine, histamine), Fos-positive cells were seen in the ipsilateral dorsal horn. Their distribution was similar to that reported previously in the mouse in response to these stimuli,^1,3,4,9,28,49,94^ and examples from mice stimulated with noxious heat, capsaicin, and histamine are shown in Figure 5Q–S. We have previously illustrated the distribution of Fos cells in mice treated with chloroquine.^3^ Fos^+^ cells were located mainly in the superficial laminae, with a rostrocaudal and mediolateral distribution that corresponded to the somatotopic location of the stimulus.^3^ Very few, if any, Fos cells were present on the contralateral side (Fig. 5T).

Pax2 antibody was included in the immunoreaction for this part of the study so that we could identify the excitatory (Pax2 negative) PPTA-immunoreactive neurons. However, we also took the opportunity to determine the proportion of Pax2-positive and Pax2-negative cells among the neurons that were Fos immunoreactive after each stimulus (Fig. 5, Table 3). In the experiments involving noxious heat, the proportion of Pax2-positive cells with Fos (51%) was significantly higher than the proportion of Pax2-negative cells (37%) (*P* < 0.001, Mantel–Haenszel test). For the other stimuli, the proportions of excitatory and inhibitory neurons that were Fos immunoreactive did not differ significantly.

**Table 3:**
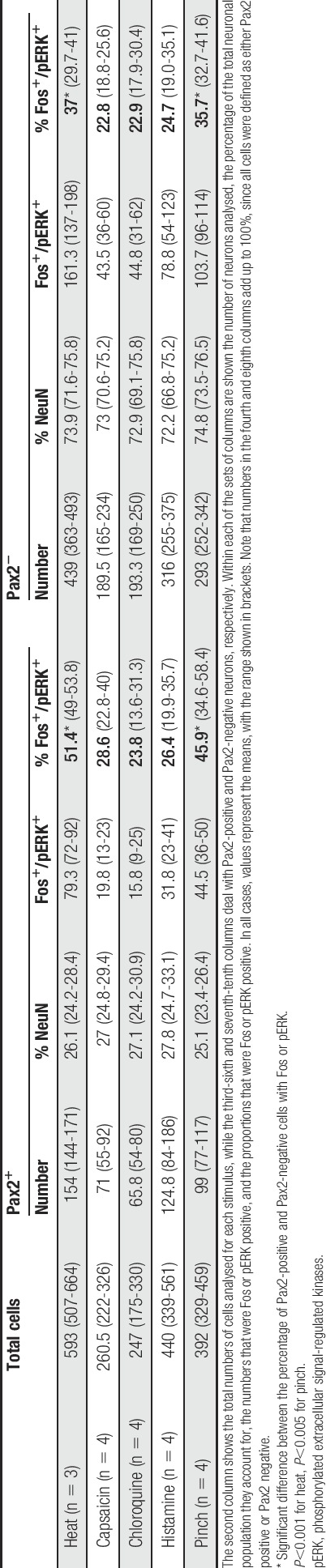
The numbers and percentages of Pax2^+^ and Pax2^−^ neurons in laminae I-II that expressed Fos or pERK after noxious or pruritic stimulation.

We then determined the proportion of excitatory (Pax2 negative) PPTA cells with Fos and compared this with the proportion of all Pax2-negative cells that were Fos immunoreactive for each stimulus (Table 4). For all the stimuli tested, some of the excitatory PPTA cells were Fos immunoreactive (Fig. 5). The proportions of excitatory PPTA cells with Fos varied between ∼30% and 40% for noxious heat and the 2 pruritogens but was considerably lower for capsaicin (9%). When we compared these values with the proportions of all excitatory neurons in laminae I-II that showed Fos, we found that they differed significantly for the mice that had been injected with capsaicin or histamine. In the capsaicin-injected mice, PPTA cells were significantly underrepresented (9% Fos immunoreactive) compared with all excitatory neurons (23% Fos immunoreactive) (*P* < 0.005, Mantel–Haenszel test). In contrast, PPTA cells were overrepresented among the excitatory neurons that were Fos immunoreactive after histamine (38% for PPTA cells, 25% for all excitatory neurons; *P* < 0.001, Mantel–Haenszel test).

**Table 4:**
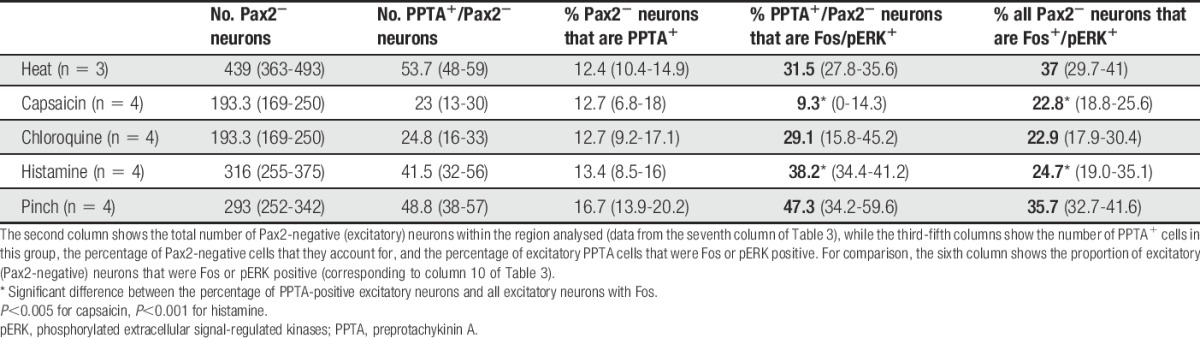
The numbers and percentages of excitatory PPTA-immunoreactive neurons in laminae I-II that express Fos or pERK after noxious or pruritic stimulation.

**Figure 6.**
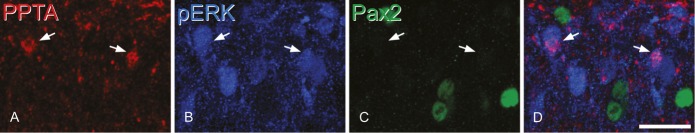
Phosphorylated extracellular signal-regulated kinases (pERK) immunoreactivity after pinch. (A–C) Show immunostaining for preprotachykinin A (PPTA) (red), pERK (blue), and Pax2 (green) in the superficial dorsal horn of an animal that was perfusion fixed 5 minutes after pinching of the calf under urethane anaesthesia, while D shows a merged image. Two PPTA-immunoreactive cells that lack Pax2 are pERK positive (arrows). Images are projections of 3 optical sections at 1 μm z-spacing. Scale bar = 20 μm.

After pinching of the skin, the distribution of pERK^+^ cells was similar to that described previously.^36,72^ The proportions of Pax2^+^ and Pax^−^ cells that were pERK immunoreactive within the activated region were 46% and 36%, respectively (Table 3), and this differed significantly (*P* < 0.005, Mantel–Haenszel test). Among the excitatory PPTA cells, 47% were pERK immunoreactive (Fig. 6, Table 4), although the difference between this proportion and the proportion of all excitatory neurons with pERK did not reach significance.

Together, these results show that inhibitory neurons in laminae I-II were more likely than excitatory neurons to show Fos or pERK following the noxious mechanical or heat stimuli and that among the excitatory neurons PPTA cells were overrepresented following histamine but underrepresented after capsaicin. It should be noted that neurons may respond to these stimuli without expressing Fos or phosphorylating ERK, and therefore, the numbers of cells revealed with these markers are almost certainly an underestimate of the numbers activated. However, these results suggest that the PPTA cells were more effectively activated by histamine, and less by capsaicin, than other excitatory neurons in this region.

### 3.4. Injection of AAV.flex.eGFP into Tac1^Cre^;Ai9 mice

**Figure 7.**
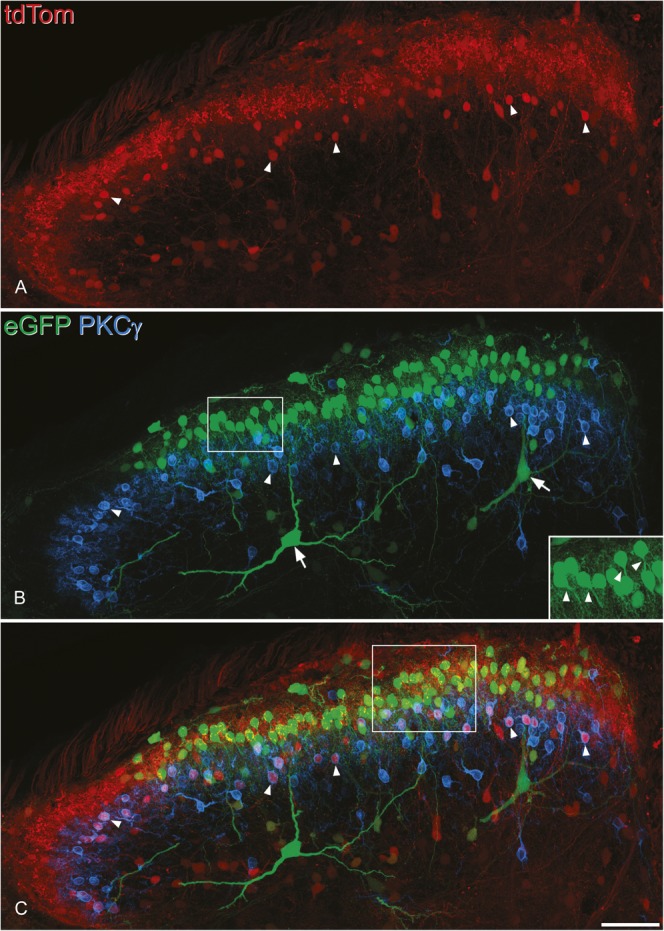
The distribution of tdTomato (tdTom)- and enhanced green fluorescent protein (eGFP)–positive cells in the dorsal horn after intraspinal injection of AAV.flex.eGFP into a Tac1^Cre^;Ai9 mouse. The section has been scanned to reveal tdTom (red), eGFP (green), and protein kinase C (PKC) γ (blue). (A) The TdTom^+^ neurons are concentrated in the superficial laminae and scattered through the deep dorsal horn. (B) The distribution of eGFP^+^ neurons is more restricted, as most of these lie dorsal to the band of neurons immunoreactive for PKCγ, which occupy lamina IIi. Note that none of the eGFP^+^ cells are PKCγ immunoreactive. In addition, 2 very large eGFP^+^ neurons (arrows) are located in laminae III-IV, and both have dendrites that extend into the superficial laminae. The inset (corresponding to the box) shows some of the eGFP^+^ cells at higher magnification, and primary dendrites can be seen leaving the ventral surface of the soma in several cases (arrowheads). (C) In the merged image, it can be seen that there are many tdTom^+^ neurons that lack eGFP (and therefore appear red) and that these include PKCγ-immunoreactive cells (some of these indicated with arrowheads). The images are projected from 45 optical sections at 1 μm z-spacing. The box in (C) indicates the region shown in Figure 8. Scale bar = 50 μm.

**Figure 8.**
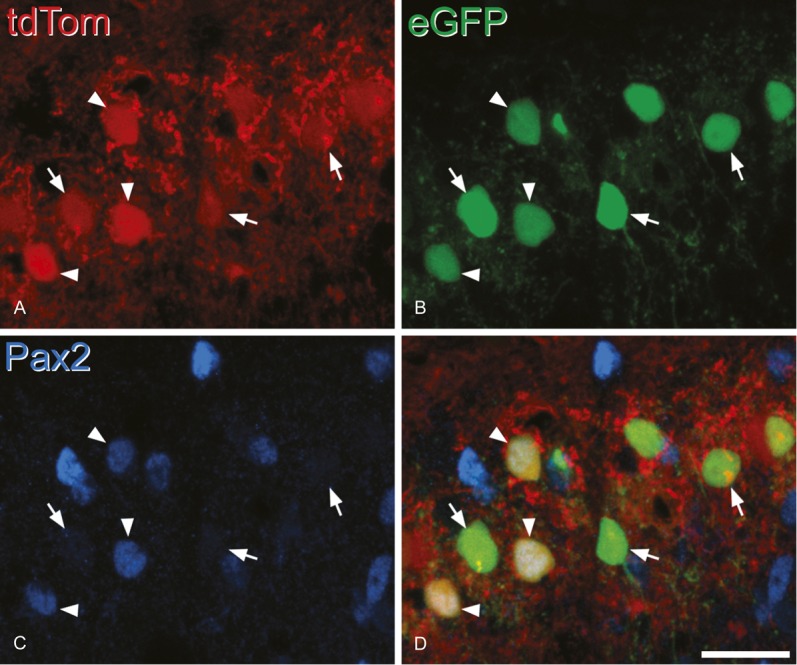
Pax2-positive and negative enhanced green fluorescent protein (eGFP)–expressing neurons after intraspinal injection of AAV.flex.eGFP into a Tac1^Cre^;Ai9 mouse. Part of the section shown in Figure 7 (corresponding to the box in Fig. 7C) scanned to reveal: (A) tdTomato (tdTom) (red), (B) eGFP (green), and (C) Pax2 (blue). (D) Shows a merged image. Several eGFP^+^ cells are visible, and all these are tdTom^+^. Some of these cells have Pax2 immunoreactivity in their nuclei (arrowheads), while others are Pax2 negative (3 indicated with arrows). This is a projection of 7 optical sections at 1 μm z-spacing. Note that the difference in appearance between the image in (D) and that seen in Figure 7C is because this shows a more limited number of optical sections, and in addition, the blue colour represents Pax2 here, but protein kinase C gamma in Figure 7C. Scale bar = 20 μm.

**Figure 9.**
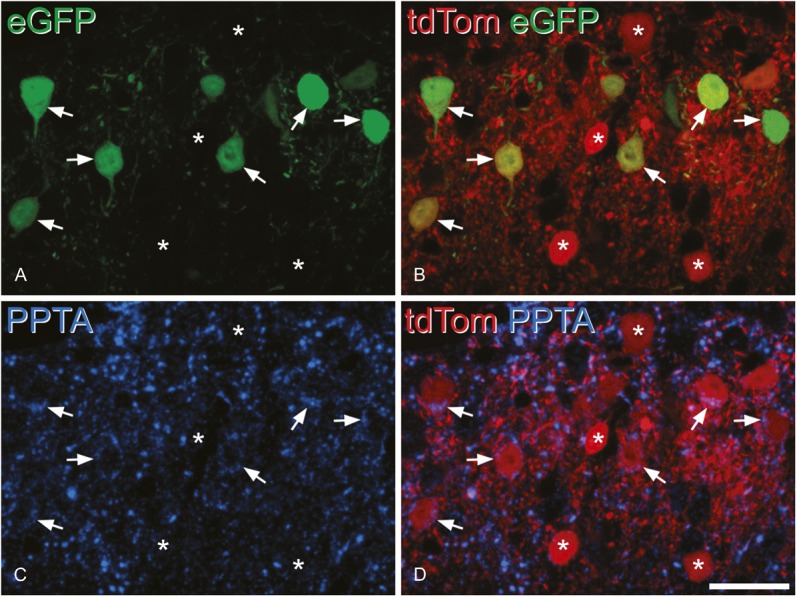
Preprotachykinin A (PPTA) immunoreactivity in enhanced green fluorescent protein (eGFP)–expressing neurons after intraspinal injection of AAV.flex.eGFP into a Tac1^Cre^;Ai9 mouse. The section has been scanned to reveal eGFP (green), tdTomato (tdTom) (red), and PPTA immunoreactivity (blue). (A) This field shows several eGFP^+^ neurons, and some are indicated with arrows. (B) All the eGFP^+^ neurons also express tdTom, and there are additional tdTom^+^ cells that lack eGFP (some marked with asterisks). (C) Shows PPTA immunoreactivity in the same field, and in (D), this has been merged with tdTom. PPTA can be seen in some of the cells marked with arrows (ie, those that are also eGFP^+^). It is present in the form of clumps within the perikaryal cytoplasm, where it overlaps the tdTom. Note that the tdTom^+^/eGFP^−^ neurons are not PPTA immunoreactive. The section is a projection of 3 optical sections at 0.5 μm z-spacing. Scale bar = 20 μm.

TdTom-positive cells were present throughout the dorsal horn in the Tac1^Cre^;Ai9 mice, with the highest concentration in the superficial laminae (Fig. 7A). Injection of AAV.flex.eGFP resulted in widespread expression of eGFP at the segmental level of the injection sites. The eGFP-positive cells were particularly numerous in the outer part of lamina II (IIo), with a few cells in lamina I or scattered through the deeper dorsal horn (Fig. 7B). The eGFP cells were present in the medial half to two-thirds of the dorsal horn, and this distribution presumably reflects the location of the injection sites. Some of the eGFP^+^ cells in lamina III had dendrites that extended dorsally into the superficial region (Fig. 7B), resembling anterolateral tract projection neurons.^7,55,80^ Primary dendrites of the eGFP cells in lamina II often emerged from the ventral aspect of the soma (Fig. 7B inset), although these could not be followed far because of the density of eGFP-labelled profiles in this region. The total numbers of lamina I-II neurons analysed in the disector sample for the 2 mice were 184 and 329, and 28% of these (27.1% and 28.8%, respectively) were tdTom^+^. Within the zone occupied by GFP^+^ cells, these accounted for 19.6% (21.2%, 17.9%) of all lamina I-II neurons, and virtually all these (96%) were also tdTom^+^ (Figs. 8 and 9). Within this region, eGFP was therefore detected in ∼70% of the tdTom^+^ neurons.

In sections stained for PKCγ and Pax2, we noted that the band of eGFP^+^ neurons lay dorsal to that of the PKCγ cells and that although many of the PKCγ cells were labelled with tdTom they were very seldom eGFP^+^ (Fig. 7B and C). Quantitative analysis confirmed this, since we found that only 9 out of 363 eGFP cells examined (2.5%) were PKCγ immunoreactive. In fact, 6 of these cells were Pax2 positive, and therefore, only 3/363 (0.8%) of the eGFP^+^ cells belonged to the excitatory (Pax2 negative) PKCγ population (Table 5). This is consistent with the lack of colocalisation between PPTA and PKCγ immunoreactivities, described above. In contrast, 44% of the tdTom^+^ cells that lacked eGFP were PKCγ neurons (Fig. 7C, Table 5). Also, consistent with the finding that some PPTA-immunoreactive neurons are Pax2^+^, we found that 11% of eGFP^+^ cells were Pax2 immunoreactive, as were 9% of eGFP^−^/tdTom^+^ cells (Fig. 8, Table 5).

**Table 5:**
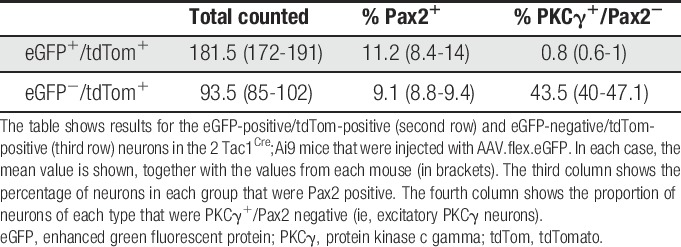
PKCγ and Pax2 expression by eGFP- and tdTom-labelled neurons in the Tac1^Cre^;Ai9 mice injected with AAV.flex.eGFP.

In the sections immunostained for PPTA, we identified 100 PPTA-immunoreactive tdTom^+^ cells, and all but one of these (99%) were positive for eGFP (Fig. 9).

Based on the proportion of neurons in laminae I-II that were eGFP^+^ (∼20%) and the finding that 90% of these were Pax2 negative, we estimate that the eGFP^+^ excitatory cells account for 18% of all neurons in laminae I-II. We have previously estimated that excitatory neurons make up 74.2% of the total neuronal population in these laminae,^60^ and therefore, the eGFP^+^ cells would correspond to ∼24% of these excitatory neurons. The difference between this value and the ∼15% of excitatory neurons that are PPTA immunoreactive (see above) could result from a low level of somatic PPTA in some cells, meaning that immunocytochemistry for PPTA underestimates the proportion of excitatory neurons that express PPTA and therefore release SP. Alternatively, some of the cells with eGFP may fail to produce significant amounts of PPTA, eg, due to degradation of the mRNA.^84^ In either case, it is likely that the proportion of excitatory neurons in laminae I-II that express PPTA and release SP is between 15% and 24%.

## 4. Discussion

The main findings of this study are that (1) PPTA can be detected in ∼20% of neurons in laminae I-II, most of which are Pax2 negative, and so presumably excitatory; (2) these are quite distinct from populations defined by expression of GRP, neurotensin, NKB, or PKCγ, but many of them contain somatostatin; (3) many of the excitatory PPTA cells respond to noxious or pruritic stimuli; and (4) this population can also be detected by intraspinal injection of AAV.flex.eGFP in adult Tac1^Cre^ mice.

### 4.1. Substance P–expressing dorsal horn neurons

Several immunocytochemical studies have identified SP-containing neurons in laminae I-II with a distribution similar to that reported here for PPTA.^33,34,43,54,68,71,92^ However, in these studies, colchicine was used to increase the concentration of peptide in the cell bodies, and this approach has the potential problem that colchicine may alter mRNA levels and even result in de novo synthesis of neuropeptides.^12,67^ A recent in situ hybridisation study^88^ identified a “late wave” of Tac1 mRNA neurons in laminae I-II that were dependent on the expression of transcription factor Tlx3, which determines glutamatergic fate.^11^ These cells were thought to originate from the dIL_B_ population of late-born excitatory interneurons,^31^ which also give rise to several other neurochemically defined classes in the superficial laminae, including neurons that express PKCγ, somatostatin, neurotensin, NKB, GRP, and the GRP receptor.^87,88^ An advantage of using the PPTA antibody is the greater versatility of immunocytochemistry compared with in situ hybridisation, eg, the possibility of multiple labelling, which allows detailed characterisation of the cells.

Excitatory interneurons arising from the dIL_B_ population are neurochemically heterogeneous.^6,25,88^ Our previous studies,^26,27^ together with the present results, demonstrate a complex pattern of intersection of neurochemical markers. Four neuropeptides (SP, GRP, neurotensin, and NKB) are expressed in largely nonoverlapping populations, with the neurotensin- and NKB-expressing cells being mainly included among the PKCγ neurons in lamina IIi. We previously estimated that GRP and NKB cells each made up ∼15% of the excitatory neurons in laminae I-II and neurotensin cells a further 8%.^26^ The present results suggest that SP-expressing neurons account for between 15% and 24% of the excitatory neurons, meaning that together these 4 populations account for over half of the glutamatergic neurons in the superficial laminae. In contrast, somatostatin is widely expressed among the excitatory neurons and overlaps extensively with each of these neuropeptides.

The finding that some PPTA-immunoreactive cells were Pax2^+^, and therefore presumably inhibitory interneurons, was surprising for 2 reasons. First, it has been reported that superficial dorsal horn cells with Tac1 mRNA are lost in Tlx3-mutant mice,^88^ and second, we found in the rat that all nonprimary SP-immunoreactive axon terminals were VGLUT2 immunoreactive.^79^ Consistent with our findings in rat, we saw only very low levels of SP in a few inhibitory (VGAT immunoreactive) boutons in the mouse. However, our interpretation that some inhibitory interneurons express PPTA was supported by the finding of Pax2-positive eGFP cells in the Tac1^Cre^;Ai9 mice that had been injected with AAV.flex.eGFP. It is therefore likely that some inhibitory interneurons express PPTA but do not have significant quantities of SP in their axon terminals. This could result from failure to cleave the active peptide from the precursor, failure to transport it to the axon terminal, or its rapid degradation.^84^

### 4.2. Functional role of substance P–expressing excitatory interneurons in laminae I-II

Recent studies of mice in which subsets of excitatory interneurons in the superficial dorsal horn have either failed to develop^85,88^ or have been ablated^16^ indicate that these cells play a critical role in perception of both pain and itch. Our finding that the PPTA-expressing excitatory cells include many that respond to noxious or pruritic stimuli suggests that loss of these cells contributed to the reduction in pain and itch behaviours seen in these studies. Among the other neurochemical types of excitatory interneurons, those expressing neurotensin and NKB are located in laminae IIi and III and correspond largely to the PKCγ cells.^26^ They are therefore unlikely to respond to noxious or pruritic stimuli.^56^ We have shown that the GRP cells seldom show Fos or pERK after injection of chloroquine,^3^ and we find that they rarely have Fos or pERK after other noxious or pruritic stimuli (AMB and AJT, unpublished observations). Interestingly, although many PPTA^+^ cells responded to noxious thermal and mechanical stimuli, they were significantly underrepresented among those that showed Fos after injection of capsaicin, even though TRPV1 plays an important role in detection of noxious heat. This suggests that they may be preferentially innervated by nociceptors that lack the capsaicin receptor TRPV1, and these could include non-peptidergic C fibres that express the mas-related G protein–coupled receptor MrgD^10,95^ as well as myelinated afferents.^69^

Mice lacking SP or its receptor (the neurokinin 1 receptor, NK1r) show exaggerated pain,^8,15^ suggesting a role for SP in nociceptive processing. However, since ∼80% of SP in the dorsal horn originates from primary afferents,^37^ the contribution of peptide released by spinal neurons cannot yet be determined. Recent studies indicate that different populations of inhibitory interneurons are responsible for suppression of itch and pain,^41,59^ but much less is known about the roles of excitatory interneuron populations. Our finding that many PPTA neurons were activated by noxious and pruritic stimuli could result from convergence of input onto these cells, but it is possible that different subsets are involved in transmitting itch and pain, and further studies will be needed to address this. Another important issue is the relation of the PPTA cells to those that express GRP receptor, which are also dependent on Tlx3^88^ and which have been implicated in spinal itch pathways.^51,75,76^

Although the morphology of these cells is not yet known, the finding that many eGFP^+^ cells in the injected Tac1^Cre^;Ai9 mice had ventrally directed primary dendrites raises the possibility that they correspond to vertical cells.^23^ Vertical cells (also known as stalked cells^21^) form a well-defined morphological class of excitatory interneurons, which are more numerous in lamina IIo, and have prominent ventrally directed dendritic trees.^23,47,48,81,91^ They are likely to account for a relatively large proportion of the excitatory interneurons in this lamina because in a blind whole-cell patch clamp study in the rat, 12 of 33 excitatory interneurons identified could be assigned to this class.^91^ The GRP cells seldom show vertical morphology^27^ and neither do PKCγ cells^61^ (which include most of those expressing neurotensin or NKB), suggesting that vertical cells may correspond to a specific neurochemical population. Vertical cells often have axons that enter lamina I and are thought to form part of a polysynaptic pathway that relays nociceptive information to projection cells in this lamina.^47^ They may also contribute to mechanical allodynia in pathological pain states.^46,90^ If the PPTA-expressing neurons do correspond to vertical cells, this would suggest that they have a dual excitatory action on lamina I anterolateral tract projection neurons: first through glutamatergic synapses^47^ and second through SP acting on NK1rs, which are expressed by 90% of these cells.^7^

### 4.3. Targeting the substance P–expressing cells in the Tac1^Cre^ mouse line

To investigate the roles of the SP-expressing interneurons, it will be necessary to manipulate their function or ablate them, and these approaches have recently been applied to several other populations of dorsal horn neurons.^14,16,18,58,59^ The Tac1^Cre^ mouse provides a convenient way of doing this, but there are several caveats. First, PPTA is expressed by many nociceptive primary afferents, by a small number of superficial inhibitory interneurons, as well as by some lamina I and deep dorsal horn neurons that may belong to the anterolateral tract.^7^ In addition, comparison of the tdTom and eGFP cells in the injected Tac1^Cre^ mice shows that there is expression of tdTom in excitatory neurons that do not appear to express PPTA in the adult, eg, many of the PKCγ neurons. It is likely that this reflects transient expression of PPTA that is switched off during development. Crossing the Tac1^Cre^ mice with other lines that express Cre-dependent proteins would therefore result in the expression of these proteins beyond the population of PPTA-expressing superficial dorsal horn excitatory interneurons. Our finding that the majority of these cells also contain somatostatin provides a potential way of targeting them selectively, since somatostatin is not expressed by SP-containing primary afferents^32,45,83^ or NK1r^+^ projection neurons in lamina I^16^ and is largely restricted to excitatory interneurons in lamina II.^16,66^ A mouse line in which another recombinase (Flpo) has been knocked into the somatostatin locus has recently been reported,^30^ and if this is crossed with the Tac1^Cre^ line, expression of both Cre and Flpo should be largely restricted to the PPTA-expressing excitatory interneurons in laminae I-II. Transient expression by other excitatory interneurons (eg, those with PKCγ) would still represent a problem, but this could be avoided by intraspinal injection of AAVs coding for proteins whose expression was dependent on both Cre and Flpo.^17^

## Conflict of interest statement

The authors have no conflicts of interest to declare.

The work was supported by the Wellcome Trust (grant 102645), the MRC (grant MR/L003430/1), and the BBSRC (grant BB/N006119/1).
